# Duplicated gall bladder with gall bladder polyp presenting with cholecystitis: Case report with literature review

**DOI:** 10.1016/j.ijscr.2019.06.002

**Published:** 2019-06-12

**Authors:** Sardar Hassan Arif, Ihsan Salih Hussein, Ayad Ahmad Mohammed

**Affiliations:** a University of Duhok, College of Medicine, Department of Surgery, Iraq; b Duhok Directorate General of Health, Azadi Teaching Hospital, Iraq

**Keywords:** Duplicated gall bladder, Gall bladder polyp, Cholecystitis, Cholecystectomy

## Abstract

**Introduction:**

Gall bladder anomalies may be in the form of abnormalities in the shape, position, or number. It could be true duplication being completely separated or Y-shaped being united with single cystic duct.

**Presentation of case:**

A 38-year-old lady presented with frequent attacks of right hypochondrial pain for the last 4 months. Ultrasound of the abdomen showed distended gall bladder with 1.3 cm gall bladder polyp at the region of the fundus. Decision done for laparoscopic cholecystectomy. During surgery a duplicated gall bladder found with single cystic duct. Successful surgery done and the gall bladder sent for the histopathological examination which showed a benign gall bladder polyp. There were no post-operative complications and the patient discharged on the third days.

**Discussion:**

Duplicated gall bladder may be diagnosed with ultrasound or magnetic resonance cholangiopancreatography (MRCP) which is the investigation of choice however most cases diagnosed intraoperatively. It may be associated with other biliary anomalies. In some occasions a single gall bladder may be removed during the first operation and the second may be missed which require another surgery. In asymptomatic individuals with duplicated gall bladder no specific treatment is required and role of surgery is controversial.

**Conclusion:**

duplicated gall bladder is a rare finding which may be completely asymptomatic. In symptomatic patients specially if associated with gall stones, surgery is required and the surgeon must be aware of any associated biliary anomalies.

## Highlights

• Duplicated gall bladder is a very rare finding.
• Complete identification of the anatomy is required before cholecystectomy.
• Sometimes one of the gallbladders may be missed during the first surgery which may require another operation.

## 1 Introduction

Anomalies of the biliary system are not uncommon; theses may involve the biliary ducts or the gall bladder. Gall bladder anomalies may be in the form of abnormalities in the shape, position, or cystic duct. They are usually discovered during surgery for gall stone disease or other surgeries involving the biliary system of the liver [1].

Anomalies of the shape could be in the form of bilobed gall bladder in which there is a single cavity with a longitudinal septum inside and a single cystic duct, hourglass type in which a transverse septum is present, Phrygian cap in which a transverse septum is present but with smaller proximal cavity. Anomalies of the number had been reported being two in number in most cases or very rarely three, it could be true duplication being two completely separated gall bladders or Y-shaped being united with single cystic duct before joining the common hepatic duct [2].

Duplication of the gallbladder is estimated to occur in about 1:4000 live births. Such anomaly may predispose to the development of gall stones due to impaired emptying of the gallbladder [3].

The work in this case presentation has been reported in line with the SCARE criteria [4].

## 2 Case presentation

A 38-year-old lady presented with frequent attacks of right hypochondrial pain for the last 4 months, the pain was mainly at the night time and was associated with nausea, no vomiting, and no fever.

The patient had no history of any medical diseases, and the past surgical history was negative.

Ultrasound of the abdomen showed distended gall bladder with 1.3 cm gall bladder polyp at the region of the fundus.

**Fig. 1:**
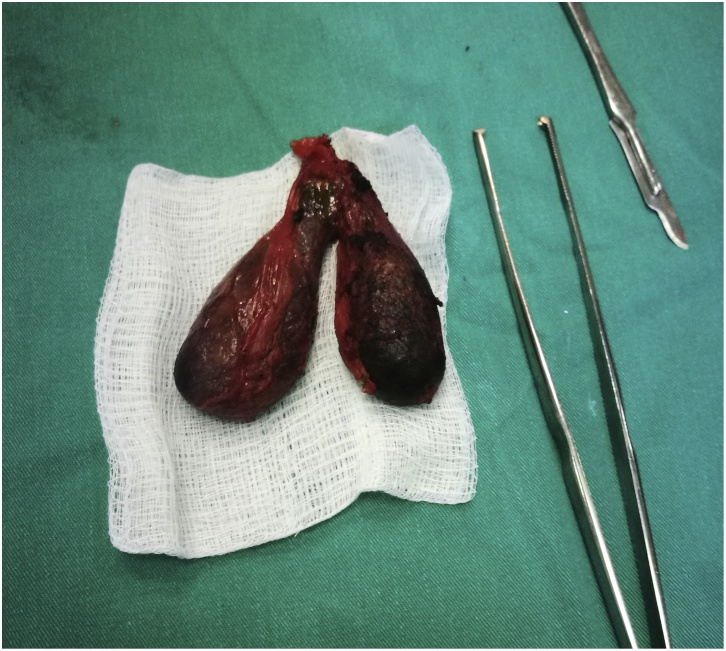
Showing the double gall bladder after being removed laparoscopically.

**Fig. 2:**
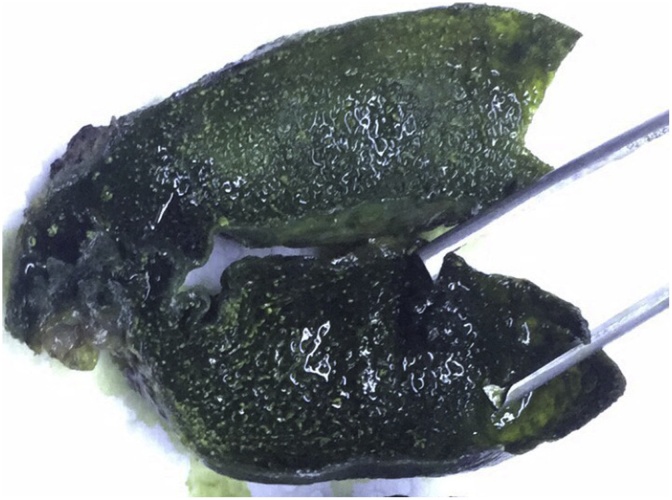
Showing the polyp arising from the mucosa of the gall bladder.

**Fig. 3:**
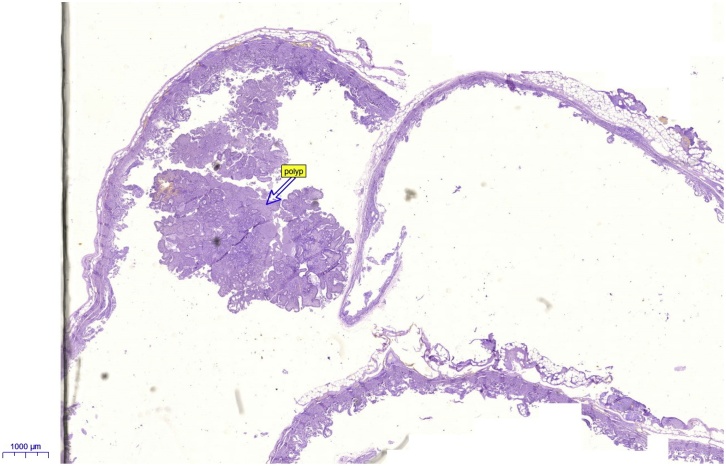
Histopathological appearance of the benign gall bladder polyp.

Advices given to the patient to reduce the fatty meals and antispasmodic medications prescribed with little improvement. Decision done for laparoscopic cholecystectomy. During surgery a duplicated gall bladder found with single cystic duct. Successful surgery done and the gall bladder sent for the histopathological examination which showed a benign gall bladder polyp (Fig. 1, Fig. 2, Fig. 3).

There were no post-operative complications and the patient discharged on the third day.

## 3 Discussion

The radiologists should be familiar with gall bladder duplication for the accurate diagnosis before surgery especially if is associated with gall stone disease. The condition can be diagnosed with ultrasound examination however magnetic resonance cholangiopancreatography (MRCP) is the investigation of choice to delineate the biliary anatomy and for the accurate preoperative plane [5,6].

Duplication of the gall bladder may be associated with duplication of cystic duct; the anatomy should be precisely visualized during surgery to avoid injuries to other structures [7].

In some rare occasions a single gall bladder may be removed during the first operation and the second one may be missed which could be diagnosed after surgery by means of MRCP or ERCP. This may mandate the surgical removal of the other gallbladder [[8], [9], [10]].

When the condition is diagnosed during surgery, intraoperative cholangiography may be required to detect any other associated biliary anomalies before proceeding with the surgical removal of the gallbladders. Sometimes the intraoperative findings may not be very clears especially if the two gall bladders are enclosed in a single peritoneal covering [11,12].

In most of the cases the operation is done with no complications although there may be some difficulties especially during dissection and delineating the anatomical structures which is very important to avoid iatrogenic injuries or missing the second one. Awareness about the presence of this condition before surgery is of utmost importance to prevent or decrease the rate of such complications [12].

The clinical presentation of duplicated gall bladder is similar to other cases which is due to stones or inflammatory process presenting as acute or chronic cholecystitis. Most authors agree in the cases of asymptomatic duplicated gall bladder requires no specific treatment and role of surgery is controversial [3].

## Conflicts of interest

The author has no conflicts of interest to declare.

## Sources of funding

None.

## Ethical approval

Ethical approval has been exempted by my institution for reporting this case.

## Consent

An informed consent is taken from the patient for publishing this case report.

## Author’s contribution

Dr Ayad Ahmad Mohammed contributed to the concept of reporting the case and the patient data recording.

Drafting the work, design, and revision done by Dr Ayad Ahmad Mohammed and Dr Sardar Hassan Arif.

Dr Ayad Ahmad Mohammed and Dr Ihsan Salih Hussein took the consent from the patient for publishing the case.

Final approval of the work to be published was done by Dr Ayad Ahmad Mohammed, Dr Ihsan Salih Hussein and Dr Sardar Hassan Arif.

## Registration of research studies

This work is case report and there is no need of registration.

## Guarantor

Dr Ayad Ahmad Mohammed is guarantor for the work.

## Provenance and peer review

Not commissioned, externally peer-reviewed.
